# 分子靶向治疗在*EGFR*突变肺鳞癌中的研究进展

**DOI:** 10.3779/j.issn.1009-3419.2024.101.07

**Published:** 2024-04-20

**Authors:** Bingwan XIONG, Wenfei KE, Wenyang JIANG

**Affiliations:** 430060 武汉，武汉大学人民医院胸外科; Department of Thoracic Surgery, Renmin Hospital of Wuhan University, Wuhan 430060, China

**Keywords:** 肺肿瘤, 表皮生长因子受体, 分子靶向治疗, 表皮生长因子受体-酪氨激酸酶抑制剂, Lung neoplasms, Epidermal growth factor receptor, Targeted therapy, Epidermal growth factor receptor-tyrosine kinase inhibitors

## Abstract

非小细胞肺癌（non-small cell lung cancer, NSCLC）是我国常见的肿瘤之一，肺鳞癌是NSCLC中除肺腺癌外最常见的组织学亚型。表皮生长因子受体（epidermal growth factor receptor, EGFR）是肺癌的驱动基因之一，相比肺腺癌，肺鳞癌中EGFR的突变率很低。针对EGFR的靶向治疗在肺腺癌中已经取得了重大进展，而在肺鳞癌中的进展相对缓慢。本文对近年EGFR突变肺鳞癌分子靶向治疗的相关研究进行综述，总结出EGFR-酪氨酸激酶抑制剂（tyrosine kinase inhibitors, TKIs）在肺鳞癌中的疗效，旨在为EGFR突变肺鳞癌患者的治疗提供参考。

## Abstract (en)

Non-small cell lung cancer (NSCLC) is a prevalent tumour type in our country, with lung squamous carcinoma being a commonly observed NSCLC subtype besides lung adenocarcinoma. Epidermal growth factor receptor (EGFR) is a significant driver gene in lung cancer, and EGFR mutation frequency is considerably lower in lung squamous carcinoma in comparison to lung adenocarcinoma. Although targeted therapy against EGFR has demonstrated significant advancements in lung adenocarcinoma, while progress in lung squamous carcinoma has been relatively sluggish. This paper reviews recent studies on molecular targeted therapy for EGFR-mutated lung squamous carcinoma and summarises the efficacy of EGFR-tyrosine kinase inhibitors (TKIs) in treating squamous carcinoma of the lung, in order to provide a reference for treating patients with EGFR-mutated squamous carcinoma of the lung.

肺癌又称原发性支气管肺癌，是一种由支气管黏膜上皮或肺泡上皮发展而来的恶性肿瘤。近年全球肺癌发病率呈明显上升趋势，20世纪末，肺癌已成为恶性肿瘤死因中的首位^[1]^。在我国被诊断为肺癌的病例中，约有85%属于非小细胞肺癌（non-small cell lung cancer, NSCLC）。在NSCLC中，肺腺癌和肺鳞癌是较为常见的两种组织学亚型^[2]^。表皮生长因子受体（epidermal growth factor receptor, EGFR）是肺腺癌中最常见的驱动基因之一，EGFR-酪氨酸激酶抑制剂（tyrosine kinase inhibitors, TKIs）在EGFR突变肺腺癌中取得了显著的疗效^[3]^。与晚期肺腺癌患者相比，肺鳞癌患者的治疗方案有限，化疗仍是标准的治疗方案^[4]^。近年来，随着分子靶向治疗的迅速发展，EGFR突变作为一种重要的治疗标志物受到了广泛关注，为肺鳞癌患者提供了新的治疗方案。研究分子靶向治疗可为EGFR突变肺鳞癌患者提供除传统化疗外的治疗方法，让部分肺鳞癌患者从中获益。

## 1 EGFR在肺鳞癌中的特点

### 1.1 EGFR与信号通路

EGFR属于HER/erbB家族，该家族包括HER1（EGFR/erbB1）、HER2（NEU/erbB2）、HER3（erbB3）和HER4（erbB4）。所有成员都具有酪氨酸激酶活性，并且表现出相似的结构：胞外配体结合区、跨膜区和胞内激酶区^[5]^。EGFR可与配体结合，产生的细胞内信号由RAS/MAPK通路、PI3K通路和STAT通路等介导，下游信号最终促进DNA复制与细胞分裂、刺激血管生成并阻断细胞凋亡^[6]^。EGFR信号通路在调控细胞生长、增殖和分化等生理过程中扮演着关键角色。但EGFR发生突变后，该信号通路持续激活，抑制肿瘤细胞的凋亡，导致其异常增殖，最终造成肿瘤的进展^[7]^。

### 1.2 EGFR突变类型

EGFR突变类型中，19号外显子缺失（19del）和21号外显子点突变（L858R）是两种最为普遍的类型，两者都为EGFR敏感突变。相比肺腺癌中高达38.0%的突变率^[8]^，EGFR在肺鳞癌中的突变率则很低。Sun等^[9]^的研究中，1359例肺鳞癌患者共出现94例EGFR突变，突变率仅为6.9%。94例患者的EGFR突变类型分别为：19号外显子突变率为37.2%（35/94）、L858R突变率为39.4%（37/94）、T790M突变率为5.3%（5/94）、G719X突变率为4.3%（4/94）、L861Q突变率为2.1%（2/94）和其他突变率为11.7%（11/94）。Memon等^[10]^发现，19号外显子区域存在2种缺失（c.2240-2254和c.2237-2251）和6种点突变。89例肺鳞癌中有50例（56.2%）患者存在c.2240-2254缺失，18例（20.2%）患者存在c.2237-2251缺失。除EGFR敏感突变外，在部分患者中还发现了一种特殊的突变类型，EGFR III型突变体（EGFR variant III, EGFR vIII）。该突变在肺鳞癌中发生的概率为5.0%-8.0%。

### 1.3 EGFR突变率与临床特征的联系

不同研究检测出的EGFR突变率存在较大差异，虽然直接测序和高通量测序对基因突变的检出率存在一定影响，但EGFR突变率的差异不能单纯用检测方法的选择来解释。实际上，EGFR突变率与患者的临床特征密切相关。Dearden等^[11]^的meta分析表明，在474例东亚肺鳞癌患者中，EGFR的突变率约为4.6%；而在334例西欧肺鳞癌患者中，EGFR突变率仅为3.3%。在其他针对亚洲人种的临床研究中，肺鳞癌患者的EGFR突变率更是高达5.4%-17.2%（表1）。值得注意的是，Memon等^[10]^的研究中，肺鳞癌患者的EGFR突变率高达86.5%，远超普通水平。研究者采用从新鲜标本中提取并反转录的cDNA作为聚合酶链式反应（polymerase chain reaction, PCR）模板，较高的PCR模板浓度和PCR-DGGE检测方法提高了检测的灵敏度。除了人种对EGFR突变率存在影响外，Zhou等^[12]^发现，女性和无吸烟史的肺鳞癌患者，其EGFR突变率明显高于男性和有吸烟史的患者（18.1% vs 5.0%, 14.3% vs 4.8%）。同时，年龄、肿瘤分化程度、临床分期、标本类型、血清癌胚抗原（carcinoembryonic antigen, CEA）水平和鳞状上皮细胞癌抗原（squamous cell carcinoma antigen, SCCAg）水平与EGFR突变率没有显著关联^[12]^。其他研究^[13,14]^指出，发生脑转移的肺鳞癌患者的EGFR突变率更高，长期油烟接触史的肺鳞癌患者的EGFR突变率更低。不过，Memon等^[10]^发现，EGFR突变率随着疾病的进展而提高，IV期肺鳞癌患者的EGFR突变率明显高于II和III期患者。

**表 1:** 随机试验中肺鳞癌的EGFR突变率和突变类型

Author	Detection methods	Mutation rate	Mutation status	Population	Year
Sun Y et al.^[9]^	PCR	6.9% (94/1359)	19del (37.2%), L858R (39.4%)	Chinese	2018
Memon AA et al.^[10]^	PCR	86.5% (77/89)	19del (88.2%)	Chinese	2017
Zhou S et al.^[12]^	PCR/NGS	8.2% (24/292)	19del (58.3%), L858R (37.5%)	Chinese	2019
Si JF et al.^[13]^	PCR/NGS	11.0% (72/657)	19del (48.6%), L858R (44.4%)	Chinese	2022
Miyamae Y et al.^[22]^	PCR	3.4% (3/87)	19del (100.0%)	Japanese	2011
Fang W et al.^[23]^	PCR	2.1% (3/146)	L858R (100.0%)	Chinese	2013
Hata A et al.^[24]^	PCR	13.3% (33/249)	19del (58.0%), L858R (36.0%)	Japanese	2014
Zhang Q et al.^[25]^	ARMS	17.2% (28/163)	19del (57.1%), L858R (39.3%)	Chinese	2015
Song Z et al.^[26]^	PCR	5.4% (4/74)	19del (50.0%), L858R (50.0%)	Chinese	2015
Tao D et al. ^[27]^	PCR	3.2% (5/157)	NA	Chinese	2016
Xu J et al.^[28]^	PCR	14.8% (33/223)	19del (51.5%), L858R (36.4%)	Chinese	2016
Joshi A et al. ^[29]^	PCR	4.5% (29/639)	19del (51.7%)	Indian	2017

EGFR: epidermal growth factor receptor; NGS: next generation sequencing; ARMS: amplification refractory mutation system; PCR: polymerase chain reaction; NA: not available.

综上，具有女性、无吸烟史和亚洲人种等特征的患者，发生EGFR突变的概率更高。但肿瘤的临床分期等其他特征与EGFR突变率是否存在关联，仍然存在争议。需要进一步的临床研究来明确突变高危人群的临床和病理特征，以指导患者的后续治疗。

### 1.4 EGFR基因检测

目前尚没有可靠的临床研究结果证实EGFR-TKIs在晚期EGFR突变肺鳞癌患者中有明显的疗效优势，故肺鳞癌患者是否需要进行EGFR基因检测也存在争议。但考虑到某些肺鳞癌患者（女性、不吸烟者、亚洲人种等）发生EGFR突变的可能性显著增加，在这些肺鳞癌患者中也应常规进行EGFR基因检测。2023年发布的中华医学会肺癌临床诊疗指南^[15]^指出，由于肿瘤的异质性，经过小标本活检诊断的肺鳞癌可能存在未被检测到的混合腺癌成分。因此对于不吸烟、经小标本活检诊断或混合腺癌成分的肺鳞癌患者，建议进行EGFR基因检测。2024版美国国立综合癌症网络（National Comprehensive Cancer Network, NCCN）指南则建议对所有肺鳞癌患者进行EGFR基因检测^[16]^。欧洲肿瘤内科学会（European Society for Medical Oncology, ESMO）建议对无吸烟史、曾有长期吸烟史（戒烟>15年）或轻度吸烟史（<15包/年）的肺鳞癌患者进行EGFR基因检测^[17]^。

## 2 EGFR-TKIs在肺鳞癌中的应用

### 2.1 EGFR-TKIs及其作用机制

EGFR-TKIs作为小分子直接进入细胞内，与EGFR催化结构域的酪氨酸激酶结合，竞争性抑制三磷酸腺苷与酪氨酸激酶结合。酪氨酸残基的磷酸化修饰和下游信号的传导受到了阻断，从而产生了抑制肿瘤生长的效果^[18]^。EGFR-TKIs包括三代药物，不同药物的作用模式有所差异。第一代EGFR-TKIs（如厄洛替尼、吉非替尼和埃克替尼）可逆地与EGFR结合，减少其二聚体自身的磷酸化，阻断细胞内信号传导^[19]^。第二代EGFR-TKIs（如阿法替尼和达可替尼）与EGFR不可逆地结合，并作用于多靶点，使所有ErbB/HER家族同源或异二聚体的信号传导受到抑制^[20]^。第三代EGFR-TKIs（如奥希替尼）选择性抑制EGFR突变，可以靶向T790M，相比第二代，副作用小且安全^[21]^。

### 2.2 EGFR-TKIs在肺鳞癌中的疗效

#### 2.2.1 第一代EGFR-TKIs

Si等^[13]^的研究发现，在接受埃克替尼治疗的101例肺鳞癌患者中，相比EGFR野生型患者，EGFR突变型患者表现出相似的总生存期（overall survival, OS）（16.8 vs 14.7个月，P=0.472）和更长的无进展生存期（progression-free survival, PFS）（5.6 vs 1.8个月，P=0.000）。但19del和L858R两种突变在接受EGFR-TKIs治疗后，患者的PFS未表现出明显差异（5.9 vs 5.6个月，P=0.376）；接受一线治疗或二线及以上治疗的患者的PFS也没有明显的统计学差异（5.1 vs 7.4个月，P=0.311）。Xu等^[28]^发现，与未接受靶向治疗的肺鳞癌患者相比，一代EGFR-TKIs显著延长了EGFR突变患者的OS（18.0 vs 13.2个月，P=0.150），但野生型患者的OS并未得到明显改善（14.0 vs 13.6个月，P=0.927）。Fang等^[23]^发现，在接受吉非替尼和厄洛替尼作为二、三线治疗的晚期肺鳞癌患者中，发生EGFR突变的患者表现出比野生型患者更好的客观缓解率（objective response rate, ORR）（26.7% vs 2.1%, P=0.002）、疾病控制率（disease control rate, DCR）（66.7% vs 41.7%, P=0.090）和PFS（3.9 vs 1.9个月，P=0.190）。Zhuang等^[30]^的meta分析总结出，接受第一代EGFR-TKIs治疗的伴有EGFR突变的晚期肺鳞癌患者，其ORR、DCR和PFS分别为31.6%、72.0%和3.1个月。另一项包含1781例晚期肺鳞癌患者的meta分析^[31]^纳入了8个随机对照试验（2个一线，6个二线），其结果显示：与安慰剂组相比，EGFR-TKIs显著延长了未经选择的肺鳞癌患者的OS和PFS。Zhou等^[12]^的研究中，EGFR-TKIs表现出比化疗更好的耐受性和更低的毒副作用。总的来说，相比EGFR野生型患者，EGFR-TKIs作为EGFR突变肺鳞癌患者的一线及二线治疗都表现出更好的疗效。

一些临床研究还对比了第一代EGFR-TKIs药物间的疗效差异。Song等^[26]^比较了吉非替尼和厄洛替尼在肺鳞癌中的疗效，结果显示吉非替尼和厄洛替尼的PFS无统计学差异（2.0 vs 1.9个月，P=0.760）。Liu等^[32]^的研究中，厄洛替尼和吉非替尼的PFS（7.0 vs 4.4个月，P=0.116）无统计学差异，但厄洛替尼组的OS（24.0 vs 11.6个月，P=0.047）更长。

不过，第一代EGFR-TKIs的局限性也非常明显，其在肺鳞癌中的疗效远不如肺腺癌。Liu等^[32]^的研究纳入了EGFR突变的肺鳞癌患者和肺腺癌患者各44例，他们分别接受EGFR-TKIs治疗（19例一线治疗，25例二线及以上治疗）。结果显示肺鳞癌组和肺腺癌组的ORR相似（43.2% vs 54.5%, P=0.290），但肺鳞癌患者DCR更低（71.3% vs 100.0%, P=0.001），PFS（5.1 vs 13.0个月，P=0.000）和OS（17.2 vs 23.6个月，P=0.027）显著缩短^[32]^。其他研究^[29,33]^也得出了相似的结论。同时，在EGFR突变肺鳞癌人群中，EGFR-TKIs并不能表现出相比化疗更好的疗效。在Si等^[13]^的研究中，接受靶向治疗的116例患者和接受非靶向治疗的541例患者的OS无明显差异（16.2 vs 13.7个月，P=0.839）。

#### 2.2.2 第二代EGFR-TKIs

LUX-Lung 8试验^[34]^对比了厄洛替尼和阿法替尼分别作为晚期肺鳞癌患者二线治疗的效果。结果显示，相比厄洛替尼，阿法替尼显著改善了肺鳞癌患者的OS（7.8 vs 6.8个月，P=0.019）。此外，阿法替尼呈现出比预期更小的毒副作用。遗憾的是，该研究中的大多数肺鳞癌患者都是EGFR野生型。是否存在潜在的预测生物标志物帮助筛选出从中获益的患者，仍是一个待解决的问题。同时，阿法替尼获得了美国食品药品监督管理局批准，用于局部晚期或转移性肺鳞癌患者的二线治疗。

#### 2.2.3 第三代EGFR-TKIs

Rekowska等^[6]^报道了1例L858R突变的晚期肺鳞癌患者，在接受奥希替尼一线治疗3个月后，疾病出现明显进展且癌症出现转移。患者对奥希替尼产生了原发性耐药，但并未对该患者进行基因检测来探查耐药产生的具体原因。Shoji等^[35]^将奥希替尼作为2例肺鳞癌患者的一线治疗，与第一代EGFR-TKIs相比，其延长了患者的PFS。Peng等^[36]^报道了1例19del的肺鳞癌患者，由于该患者发生T790M突变，导致第一代和第二代EGFR-TKIs失去疗效。患者在接受奥希替尼治疗3个月后获得病理学完全缓解。目前，关于奥希替尼在肺鳞癌中的应用很少。且上述几例病例报告中，其疗效表现出很大差异，可能与用药后是否发生耐药性有关。奥希替尼对EGFR突变肺鳞癌患者的疗效尚不明确，需要开展进一步的临床研究以评估。

综上，EGFR-TKIs对肺鳞癌的疗效在不同的回顾性临床试验中有所差异（表2），这可能与样本量、基因检测方法、药物选择和患者临床及病理特征等多种因素相关。为进一步明确EGFR-TKIs在肺鳞癌中的疗效，需要开展一项大样本的前瞻性临床试验。EGFR-TKIs对EGFR突变的肺鳞癌患者有一定疗效，可以延长患者的PFS。但相比肺腺癌，这种疗效并不理想。当前，晚期肺鳞癌患者仍然以铂类药物为主要基础的化疗方案作为标准治疗。相比之下，将EGFR-TKIs作为一线治疗方案，并未表现出优于化疗的疗效。不过某些特定患者仍可从EGFR-TKIs治疗中获益，这表明患者的临床特征对疗效存在影响。所以，有必要对不同特征的肺鳞癌患者开展对照试验，以验证EGFR-TKIs在不同亚组中疗效的差异，并筛选出从治疗中获益的人群。同时，考虑到EGFR-TKIs的低毒性和更好的耐受性，我们仍可将其作为不符合化疗条件患者的一线治疗方案以及化疗失败后的二线治疗方案。

**表 2:** EGFR-TKIs在携带EGFR突变肺鳞癌中的疗效

Items	Zhou S et al.^[12]^	Si JF et al.^[13]^	Chang Q et al.^[14]^	Fang W et al.^[23]^	Hata A et al.^[24]^	Song Z et al.^[26]^	Xu J et al.^[28]^	Joshi A et al.^[29]^	Liu Y et al.^[32]^	Liang S et al.^[33]^	Jin R et al.^[37]^
Drugs											
Gefitinib/Erlotinib	4/0	NA	16/6	15	18/2	4	7/11	9/10	26/14	0	6/3
Icotinib	8	NA	14	0	0	0	4	0	4	78	14
Afatinib/Osimertinib	2/0	NA	2/0	0	0	0	0	0	0	0	3/1
Teatment lines											
First	10	30	17	0	NA	0	NA	7	19	NA	NA
Second/Third	3/1	21	21	15	NA	4	NA	12	25	NA	NA
Mutation status											
19del	9	29	19	13	NA	2	10	NA	28	35	18
L858R	5	22	18	2	NA	2	10	NA	15	26	6
Others	0	0	1	0	NA	0	2	NA	1	17	4
ORR	28.6%	11.8%	44.7%	26.7%	25.0%	NA	31.8%	26.3%	43.2%	48.0%	NA
PFS	4.7	5.6	8.0	3.9	1.4	8.0	3.9	5.0	5.1	12.7	4.6
OS	10.6	16.8	24.9	NA	14.6	NA	18.4	13.5	17.2	18.5	NA

TKI: tyrosine kinase inhibitor; ORR: objective response rate; PFS: progression-free survival; OS: overall survival.

### 2.3 EGFR-TKIs耐药性的产生

EGFR-TKIs耐药性的产生是导致其治疗缓解率降低的主要因素^[38]^。第一、二代EGFR-TKIs产生耐药性的主要机制是T790M突变^[39]^。T790M突变后，空间位阻改变，三磷酸腺苷和EGFR的结合亲和力增强。因此，TKIs竞争性抑制EGFR的效果受到影响，导致其疗效降低^[40]^。为解决耐药性的问题，研发了可靶向T790M的第三代EGFR-TKIs。不过在使用奥希替尼治疗后，耐药性也不可避免地发生了。第三代EGFR-TKIs的耐药机制非常复杂，且在不同患者不同部位都存在异质性^[41]^。大部分肺鳞癌患者在接受EGFR-TKIs治疗失败后并未进行再次活检，针对肺鳞癌耐药性产生机制的研究很少。一项小样本研究^[14]^中，8例经过EGFR-TKIs治疗后的晚期肺鳞癌患者出现了2例T790M突变，1例MET扩增。由于样本量太小，参考价值有限。需要一项大样本量的研究，来确定EGFR-TKIs在肺鳞癌中耐药性产生的机制，并比较肺鳞癌与肺腺癌耐药性产生的差异。

## 3 影响EGFR突变肺鳞癌患者治疗方案选择的因素

组织学是影响药物疗效不容忽视的因素。组织病理学显示部分EGFR突变的肺鳞癌患者存在混合型腺癌成分^[42]^，EGFR-TKIs在这些患者中的敏感性可能取决于腺癌成分在整个肿瘤中所占的比例。女性、无吸烟史、高CEA和甲状腺转录因子-1（thyroid transcription factor-1, TTF-1）阳性等因素，都可能有腺癌倾向。我们可以考虑将EGFR-TKIs作为这些患者的一线治疗方案，可能会收获良好的临床效益。一项研究^[14]^表明，EGFR突变的女性肺鳞癌患者接受EGFR-TKIs治疗后，表现出比化疗更好的PFS（8.0 vs 3.2个月，P=0.020）和OS（24.9 vs 13.9个月，P=0.020）。Hata等^[24]^发现，5例TTF-1阳性的EGFR突变肺鳞癌患者在接受EGFR-TKIs治疗后，有4例获得了临床益处（完全缓解、部分缓解或稳定），获益率为80%。相反，对男性、目前吸烟、高细胞角蛋白19片段和p63阳性的患者，EGFR-TKIs的疗效可能并不理想，考虑化疗作为一线治疗更好。

在未经选择的肺鳞癌患者或野生型肺鳞癌患者中，应首选化疗作为治疗方案，EGFR野生型患者并不能从EGFR-TKIs中获益。此外，EGFR突变亚型也会造成TKIs疗效的差异。例如，EGFR-TKIs对G719X等罕见突变的敏感性似乎不如19del及L858R等敏感突变有效^[43]^。在这种情况下，选择化疗作为一线药物可能有更好的疗效。EGFR vIII突变对可逆性TKIs不敏感，可选择阿法替尼等不可逆性TKIs作为治疗药物。

## 4 EGFR-TKIs在肺鳞癌中疗效低的原因

EGFR-TKIs在肺腺癌中的治疗效果明显优于肺鳞癌，两者疗效差异的机制尚未阐释清楚。现有的研究认为，EGFR-TKIs在肺鳞癌中的低疗效可能与肺鳞癌特殊通路激活、特殊耐药性的产生有关。随着基因测序技术的发展，肺鳞癌基因组得到了全面细致的分析，大量的研究证实了肺鳞癌核心细胞通路上存在复杂的基因组变化，且肺鳞癌与肺腺癌基因组图谱存在明显差异^[44]^。例如，TP53和PIK3CA是肺鳞癌中显著突变的基因，其突变频率高于肺腺癌^[45]^。69.0%的肺鳞癌患者存在PI3K/RTK/RAS信号通路的改变，这些复杂通路的改变有助于解释EGFR-TKIs的低疗效。因此，建议在治疗前对KRAS、PI3KCA、MET和非致敏的EGFR突变进行联合检测。Jin等^[37]^的研究发现其他通路的突变会影响EGFR突变肺鳞癌患者的预后。其中CREBBP、ZNF217和Wnt通路突变的患者，其PFS更低；而GRM8基因突变的患者，其PFS更高。

同时，其他信号通路的激活，如ERBB2和PIK3CA可能介导了肺鳞癌对EGFR靶向治疗的耐药性^[46]^。Wang等^[47]^发现，携带EGFR突变的肺鳞癌患者对EGFR-TKIs产生耐药性是由于BMP-BMPR-Smad1/5-p70S6K激活所致，联合应用EGFR-TKIs和BMP信号通路抑制剂可克服耐药性。Ju等^[48]^发现，ILK基因敲除可以提高厄洛替尼对肺鳞癌的疗效。在肺鳞癌的发生和发展过程中，多条信号通路被激活，而肺腺癌可能依赖于单一关键通路（如EGFR、ALK或ROS-1）。因此可推测，当使用EGFR-TKIs阻断单一通路治疗时，肺鳞癌患者的疗效较差。Memon等^[10]^发现，突变的EGFR mRNA在IV期肺鳞癌患者中的比例（37.5%）显著高于II期（19.0%）和III期患者（16.6%）。Fang等^[23]^发现，肺鳞癌患者中突变的EGFR mRNA占总EGFR mRNA的比例从1.0%到100.0%不等，这也可能造成EGFR-TKIs疗效的差异。

## 5 总结与展望

在肺鳞癌患者中，EGFR突变的发生率较低，因此这类患者的治疗选择也受到限制。化疗仍是普遍的一线治疗方案，然而晚期肺鳞癌在传统的化疗方案中获益有限。当前，随着分子靶向治疗技术的迅速发展，肺鳞癌患者的治疗方案得到了重大的更新。EGFR-TKIs在部分肺鳞癌患者中已经得到了应用，为这一肺癌亚型的治疗带来了新的希望。大量的临床研究已证实，EGFR-TKIs能一定程度地改善EGFR突变肺鳞癌患者的生存期和生活质量。虽然相比肺腺癌，肺鳞癌患者使用EGFR-TKIs治疗取得的效果并不显著，但其在某些特定的肺鳞癌患者（亚洲人种、女性、无吸烟史等）中甚至表现出优于化疗的疗效。故可考虑将EGFR-TKIs作为这些患者的一线治疗方案，同时不符合化疗条件或化疗失败的患者也可考虑将EGFR-TKIs作为二线治疗方案。为进一步改善EGFR突变型肺鳞癌患者的预后，提高其生存率，仍需为晚期患者开发新的靶向治疗方案。同时根据患者自身情况，如何实行靶向治疗联合化疗或免疫疗法的个体化治疗，并确定治疗的最佳顺序，是今后研究的重点。
